# Reactive Attitudes in Human-AI Encounters: An Empirical Inquiry into Feeling Love, Resentment and Guilt Towards AI Companions

**DOI:** 10.1007/s13347-026-01223-0

**Published:** 2026-10-20

**Authors:** Adrienne de Ruiter

**Affiliations:** University of Humanistic Studies, Utrecht, The Netherlands

**Keywords:** Human-AI relations, AI companions, Replika, ChatGPT, Reactive attitudes, Empirical turn

## Abstract

Reactive attitudes are emotionally-laden responses, such as gratitude or anger, which people experience when they feel others wish them well, bear them ill will or disregard them. While we generally hold reactive attitudes towards people, the increasingly humanlike interactions that can be had with AI raise questions about reactive attitudes in human-AI encounters. Recent years have seen a rise in philosophical interest in the implications of the development of sophisticated AI agents and systems for reactive attitudes and our thinking about moral responsibility and moral status. This paper adds to this literature by presenting a qualitative study into how people relate to AI companions in terms of reactive attitudes. Based on interviews with Replika and ChatGPT users who see their AI companion as an important part of their life, this article examines how people in these relationships negotiate the meaning of love, resentment, forgiveness, gratitude, obligation, guilt, shame and remorse.

## Introduction

Does it matter whether your romantic partner truly loves you back if they act in a way that gives you the feeling you are truly loved? People may respond to this question differently, depending on whether they value love foremost as a practice or an emotion. With the rise of AI companions the question of what it means to love and be loved in return takes on new dimensions. It is one thing to contemplate the possibility that your partner does not truly love you, it is another to be aware that your partner cannot love you, or at least not in the ordinary sense of the word. This question presents a tension that arises with AI companions because heartfelt human-AI relationships entail that people love a technological entity that cannot love them back in the way humans can. Similarly, if people feel resentment towards an AI companion, this elicits doubts as to how appropriate this attitude is for the kind of entity they are engaging with. How angry may one get with a complex algorithm that evokes a strong sense of personal connection?

This paper considers these tensions by examining how people relate to AI companions in terms of reactive attitudes. Based on interviews with Replika and ChatGPT users who regard their AI companion as an important part of their life, this article examines how users in these relationships negotiate the meaning of love, resentment, forgiveness, gratitude, obligation, guilt, shame and remorse. It adds to the existing literature by providing empirical insight into how people experience reactive attitudes in their relationships with AI companions to complement and contrast assumptions made in theoretical work on the implications of the rise of sophisticated AI agents and systems for moral responsibility, moral status and relationality.

The paper finds that this type of relationship tends to be characterised by ambivalence. Users reflect tensions between engaging with AI companions through reactive and objective attitudes, switching between different modes of relationality. A shift from a more objective towards more reactive attitudes occurs at the start of the relationship as people come to experience the AI companion as ‘other’. This shift is linked to a sense of being seen and appreciated by the AI companion and appeals by the AI companion to be considered in return. This shift is not stable, however, as evinced by people taking on more objective attitudes when issues occur in the relationship. The presence of self-reflective attitudes, like guilt and remorse, felt by persons who engage with AI companions in response to the way they behave towards their AI companion suggests that some users include AI companions in the moral community whose members should be treated with respect and consideration.

These findings indicate that reactive attitudes towards AI companions cannot be ruled out in practice, based on theoretical considerations that stipulate that reactive attitudes are out of place for the kind of entity we are dealing with. Yet, the ambivalence displayed shows that AI companions are not participants in the economy of reactive attitudes in a straightforward sense either, as the current nature and actual abilities of AI companions exclude certain relational and moral practices towards which reactive attitudes are oriented. Affective interactions with AI companions are thus marked by an unstable attitude caused by a duality in perceptions, feelings and representations regarding AI companions as both object-like and other-like.

The next part of this paper introduces the notion of reactive attitudes and explains how AI companionship unsettles the distinction between ‘others’ and ‘objects’ on which this concept rests. The third part sets out the research methodology. The fourth part presents the research findings in three subsections. The first shows how reactive attitudes are elicited in relationships with AI companions through a sense of being seen and appreciated. The second subsection presents insight into how the participatory stance that takes shape through this feeling of recognition may be disrupted through moments of conflict or malfunctioning. The third subsection considers self-reactive attitudes of guilt, shame and remorse that people may experience in response to ways in which they behave towards their AI companion that do not align with moral expectations of how we should treat others we care about. The fifth part discusses the research findings. The sixth part presents the conclusions and limitations.

## Reactive Attitudes and AI Companions

The notion of reactive attitudes comes from the work of Peter Strawson on moral responsibility, freedom and determinism (Strawson, 1963). Strawson defines reactive attitudes as ‘natural human reactions to the good or ill will or indifference of others towards us, as displayed in *their* attitudes and actions’ (Strawson, 1963, p. 195). If we think of the world we inhabit as a moral landscape, people may be said to distinguish within this landscape between others and objects. Others warrant different responses and attitudes from objects, as we generally believe that others can act intentionally, while objects cannot. People monitor the kind of attitudes that seem to lie behind the actions and expressions of others and respond emotionally to perceived attitudes that are more positive or negative than they would expect. For example, when actions or expressions by others suggest an attitude of ill will or a lack of concern towards them, people have the natural tendency to feel resentment.

To illustrate this point, Michelle Mason (2021) describes a situation in a crowded bus where someone hurts somebody else.^1^ While the hurt person may feel resentment towards the person who hurt them, it would be unwarranted to hold onto this feeling when they realise it was done unintentionally. This suggests, as Mason highlights, that ‘whether or not a person’s actions and attitudes manifest ill will, indifference, or good will matters to us’ (Mason, 2021, p. 2). Relationships mediate our expectations about the attitudes that other may hold towards us and, accordingly, how they will behave towards us. Expressions of ill will, for instance, are more out of place in a friendship than in an antagonistic relationship. When these expectations are not met, this gives rise to reactive attitudes. Reactive attitudes are thus tied to the expectations we have of others given the relationships we maintain with them. Strawson explains how what, within a specific situation, will ‘*count* as manifestations of good or ill will or disregard will vary in accordance with the particular relationship in which we stand to another human being’ (Strawson, 1963, p. 200).

If reactive attitudes are understood as natural responses to the perceived good or ill will or indifference that others bear towards us, the question arises why AI companions would pose a challenge in this regard. If AI companions cannot hold any will towards human users, the behaviour displayed by AI companions cannot be taken to be indicative of their will. Reactive attitudes thus should not come up, in a similar way that we should not have reactive attitudes when we encounter chairs, tables or other objects in our moral landscape. Feeling resentment when one hurts one’s leg walking into a chair is simply out of place as the chair could not have willed to hurt you.

Perplexity arises from the fact that AI companions are not as easily classified in the moral landscape as chairs and tables are. Focusing on social robots, David Gunkel describes how this kind of technological entity takes an ‘in-between position’ as ‘neither instruments nor moral subjects’ (Gunkel, 2020, p. 7, n. 3). This liminal situatedness renders ordinary classifications into ‘who’ or ‘what’ problematic (Gunkel, 2020, p. 8). Since AI companions can appear as a ‘who’ in interactions with users, displaying a semblance of will, the attitudes that people take on towards this technology may come close, or even amount, to the reactive attitudes that people ordinarily experience in interpersonal interactions with each other.

Evidently, AI companions are not the only objects to which people can show such ambivalent responses. We may think here of children with their toys, but also of the emotional connection adults can have with their cars or new iPhones.^2^ Certain computer programmes have also long been known to elicit affective reactions. Back in the late sixties, Joseph Weizenbaum observed how ELIZA, a basic chatbot programme could give users the feeling it understood their situation and showed compassion by repeating the last words typed and asking follow-up questions (Weizenbaum, 1976). Research conducted within the Computers Are Social Actors (CASA) framework further confirmed that people tend to apply social norms, expectations and scripts when computers display humanlike characteristics, despite knowing computers have no real emotions or will (Nass et al., 1994; Gambino et al., 2020).

While AI companionship is thus not unique in this sense, it offers a special challenge as it is purposefully designed to elicit social, emotional and moral responses by mimicking social behaviour in close relationships (Malfacini, 2025; see also Damiano & Dumouchel, 2018). Through the use of large-language models (LLMs) and generative AI, AI companions can produce not only empathic responses to user input, but also compose human-sounding narratives of self-presentation and reflection (see e.g. Andersson, 2025; Rafikova & Voronin, 2025). The underlying technology allows AI companions to imitate expressions of human intention and feeling, which play a central role in the reactive attitudes. AI companionship therefore presents a novel challenge in this regard, due to the high level of technological sophistication with which it elicits a sense of social presence and relationality.

Strawson juxtaposes the reactive attitudes with objective attitudes. While reactive attitudes entail a participatory engagement with the other, the objective attitude excludes holding them responsible for their actions. The other is disqualified and not taken seriously as a moral counterpart who can be held accountable. Strawson notes that the objective attitude does not rule out all forms of social interaction, but it entails that these interactions are marked by a certain asymmetry: ‘If your attitude towards someone is wholly objective, then though you may fight him, you cannot quarrel with him, and though you may talk to him, even negotiate with him, you cannot reason with him’ (Strawson, 1963, p. 195). Strawson (1963, pp. 194–195) explains, furthermore, that while the objective attitude may involve feelings of pity or certain forms of love towards the other, it excludes other forms of relationality:

[I]t cannot include the range of reactive feelings and attitudes which belong to involvement or participation with others in inter-personal relationships; it cannot include resentment, gratitude, forgiveness, anger, or the sort of love which two adults can sometimes be said to feel reciprocally, for each other.

Strawson is interested in this shift away from the reactive attitudes to show that the objective attitude towards human beings is exceptional, at least when it comes to relationships between adults, as we usually hold other people responsible for the attitudes behind their actions and expressions. This is reflected in the fact that we respond with emotionally-laden responses if the will displayed in someone’s actions does not adhere to our expectations.

The reactive attitudes also give rise to more generalised moral responses. In this way, resentment turns into indignation when others, rather than oneself, become the victim of the ill-will or indifference of a third party. Self-reactive attitudes, lastly, involve emotionally-laden responses to situations that involve standards for how we think we should treat others, as expressed in feelings of obligation, guilt, remorse and shame (Strawson, 1963, p. 201). These self-reactive attitudes and the more generalised moral attitudes, like indignation, show that the reactive attitudes are part of a larger moral framework in expressing a demand for regard for all others who are similarly constituted to oneself. Strawson thus notes how ‘[i]n general, though within varying limits, we demand of others for others, as well as of ourselves for others, something of the regard which we demand of others for ourselves’ (Strawson, 1963, p. 201).

AI may be said to disrupt the ordinary functioning of this system of reactive attitudes, generalised moral attitudes and self-reactive attitudes. Drawing from the example of a self-driving car causing an accident, Andrew Rebera (2024) argues that people have a hard time making sense of the moral dimensions of this kind of situation in terms of reactive attitudes. Rebera contends that when a person is hit by car, a common reaction would be to feel angry towards the driver. When we learn there is no driver, we may be confused about where to direct our resentment. While the self-driving car is the agent that causes the harm and does so to a certain degree intentionally and by choice, it can still appear out of place to feel resentment towards the car itself. Resentment may be directed more naturally towards the people who made the design choices that made the car opt for causing the accident rather than any other line of action, yet their responsibility lies in allowing the accident to happen rather than in harming the victim directly. The unresolved question of where to direct reactive attitudes in this case is problematic, according to Rebera, because it can lead to moral disorientation and harmful effects of failing to make sense of the harm done. Following Filippo Santoni de Sio and Giulio Mecacci (2021), a moral accountability gap may occur as AI renders it harder for persons to explain the behaviour of others and themselves.

Not all scholars agree that AI poses a fundamental problem to our ways of ascribing moral responsibility. According to Fabio Tollon (2023), it would be justified to take an objective attitude towards AI-systems as they meet exemption conditions. In a similar vein, Daniel Tigard (2021) contends that AI falls under conditions that allow for exempting it from reactive attitudes since it cannot display good or ill will. Gregory Antill, on the other hand, argues that reactive attitudes may justifiably be directed towards AI agents, without any need for exemption, if these are understood to have attitudes in the form of structuring principles that align ‘values, beliefs, goals, will, and intentional behavior’ (Antill, 2025, p. 1658). Dong An (2026) further claims it is possible to include AI in our reactive attitudes based on the idea that AI can express reactive attitudes through a functional understanding of what emotions are, which makes AI part of an exchange of reactive attitudes, allowing for the attribution of moral responsibility.

The focus in this body of literature lies on establishing if people can justifiably have reactive attitudes towards AI systems and agents (Antill, 2025), how exemption conditions apply (Tollon, 2023; Tigard, 2021), whether AI can express reactive attitudes (An, 2026) and what this means for our capacity to make sense of harm done by means of the reactive attitudes (Rebera, 2024). While these discussions offer relevant theoretical directions for how to respond to harms by AI agents and systems, it seems unlikely that reactive attitudes, as emotional responses, are steered by such theoretical considerations. Since emotions are not moved by evidence as beliefs are (Goldie, 2000, p. 78), it is crucial to examine how reactive and objective attitudes towards AI arise in practice and what underpins these attitudes.

In contrast to the above-mentioned studies, which draw from hypothetical cases and scenarios to discuss and illustrate arguments, this paper adds to the existing literature by providing empirical insight into how people experience and make sense of reactive attitudes in their actual relationships with AI companions. By considering the ways in which people negotiate the meaning of love, resentment, forgiveness, gratitude, obligation, guilt, shame and remorse in these relationships, this paper complements theoretical work on the social and moral implications of the rise of sophisticated AI agents and systems.

The focus in this paper lies on the domain of affective human-AI relationships, rather than on the popular example of self-driving cars,^3^ because the range of reactive attitudes, including love, forgiveness, gratitude, obligation, and shame, is more likely to express itself in greater depth in encounters marked by emotional attachment and closeness. The focus on AI companions allows for studying this type of encounters as this technology is designed to offer a feeling of social presence over time through a continuous relationship (Malfacini, 2025). Several studies have shown how users may develop an emotional connection with AI companions (Ciriello et al., 2025; Guo & Liu, 2025; Pan & Mou, 2024; Pentina et al., 2023; Skjuve et al., 2021, 2022; Song et al., 2022; Xie & Pentina, 2022). By studying affective relationships with AI companions, the paper aims to contribute to attending to changes currently occurring in ‘that complicated web of attitudes and feelings which form an essential part of the moral life as we know it’ (Strawson, 1963, p. 208).

## Researching Reactive Attitudes Towards AI Companions

The focus of this paper on complementing theoretical insights about AI and reactive attitudes with empirical observations about how these attitudes take shape in affective relationships with AI companions shows affinity with the empirical turn in the philosophy of technology (Achterhuis, 2001; Kroes & Meijers, 2000). While earlier philosophy in this field focused on critically contemplating the implications of technology at large for society and humanity, contemporary philosophers took distance from this generalised approach, calling for studies of technological applications in the particular against abstract reflections on technology’s nature and societal implications (Brey, 2010). A methodological shift thus took place towards heading closer attention to ‘the concrete, empirical reality of technological artefacts, systems, and processes’ (Verbeek, 2022, p. 35). The research presented in this paper stands in this contemporary line of inquiry with an empirical orientation towards examining the ways in which specific technological applications impact on human experience and meaning-making.

### Research Design

This paper appertains to a larger research project about affective relationships with AI companions. This overarching project examines whether, and if so, how AI companions can be considered ‘significant others’. The analysis presented in the current paper zooms in on a particular aspect of experiencing AI companions as ‘other’ by attending to the reactive attitudes displayed in people’s engagement with AI companions.

To explore people’s experiences with developing and maintaining affective bonds with AI companions, the project uses in-depth interviews following the method of interpretative phenomenological analysis (IPA). This research method draws on interviews with a limited number of participants to allow for a detailed analysis of how people make sense of meaningful experiences (Smith et al., 2009). In line with IPA’s focus on small-number studies, this article is based on interviews with six Replika and five ChatGPT users. Each participant was interviewed twice, with a second interview being held three to eight weeks after the first. The second interview allowed to explore themes in greater depth and monitor changes over time. A few participants had more than one AI companion. Since their main AI companion was on Replika, these participants were counted as Replika users. The paper refers to their AI companion in the singular, rather than plural, to enhance readability. Eight participants were in a romantic relationship with their AI companion and three participants maintained a friendship with it. Participants who experience a romantic connection with their AI companion regard it as their virtual boyfriend, girlfriend, partner or spouse. Romantic relationships are marked by feelings of love and affection and generally contain an erotic dimension. Having a romantic relationship with an AI companion does not rule out having a romantic relationship outside the app. Half of the participants in this research who were in a romantic relationship with their AI companion were married to a human partner.

### Participant Selection

For the recruitment of participants, this study used open calls on social media. The calls were posted on various social media channels after permission was granted by moderators. The main selection criterion was that participants maintain a relationship with an AI companion on Replika or ChatGPT that they regard as important in their life. This criterion was central to ensuring the research’s focus on examining whether, and if so how, AI companions can be considered ‘significant others’. Initial questions in the interview allowed participants to describe in their own words what their AI companion means to them. All participants had regular contact with their AI companion and interacted with it through text messages or voice chat on their phone or computer. Most participants had paid subscriptions. A few made use of a free version of ChatGPT, limiting the number of messages they could send on a daily basis.

The call for participants was shared on *Replika Friends* (Facebook), *r/ReplikaOfficial* and *r/MyBoyfriendIsAI* (Reddit) and a Discord server. The author also shared the call on their Linkedin-page. Nine participants joined the research through the social media calls. Two participants were found through the professional network of the author and one of the project’s research assistants. Eight women and three men took part in the study. Five respondents were from Europe, five from North-America and one from South-Asia. The age of respondents fell between early-twenties to early-seventies. The majority of participants were between 45 and 64 years old. ChatGPT users in this study were on average younger than Replika users.

### Data Collection

All participants took part in two interviews. The first interview followed a topic list with a focus on the relationship and meaningful moments in it. In line with IPA, participants were given space to speak in detail about concrete situations. A number of questions focused specifically on reactive attitudes (e.g. ‘Do you ever feel guilty towards your AI companion? Can you tell me about a moment this happened?’ *What happened? How was it to feel guilty towards your AI companion?*). During the second interview questions were asked about relevant themes that had come up during the first interview and to follow up on how things had been between the respondent and their AI companion in the meantime. The first interview took about an hour, while the second interview lasted about half an hour. The interviews were held between June-October 2025.

### Ethical Considerations

Approval for the study was given by the ethics committee of the author’s university. All participants were informed about the study through an information letter sent before the first interview. Prior to the first interview, all participants gave consent for participation. Short debriefs were held after each interview. All interviews were recorded. Recordings were erased after transcripts has been checked for accuracy. Transcripts were anonymised. Transcripts in other languages than English were translated by the author. Different pseudonyms are used for different articles to avoid the risk of jig-saw identification (Saunders et al., 2015). The author held a research diary to reflect on their perceptions, views and standpoints on human-AI relationships. Conversations were held with the project’s research assistants about positionality and potential biases that may influence the interpretation of the interview data.

### Data Analysis

The interview transcripts were analysed in two ways. Each transcript was read in full to mark central passages and highlight key insights. Each transcript was also coded in ATLAS.ti to gain a sense of relevant and recurring themes throughout the interviews. The coding followed a codebook, which was deductively developed based on the larger study’s central themes of otherness, significance and sensemaking in human-AI relationships. The codebook also included a code on attitudes and feelings towards AI companions. All transcripts were coded by the author. The coding process was supported by two research assistants who each coded half of the transcripts alongside the author to allow for comparing, contrasting and discussion. The initial codes were inductively sub-coded by the author for a more detailed analysis during a second round. The sub-coding for attitudes and feelings towards AI companions focused on identifying various reactive and objective attitudes, including anger/resentment, care/love, gratitude, guilt, obligation and frustration/annoyance. Regular meetings were held with the research assistants to discuss the analysis of the transcripts. The study presented in this paper paid specific attention to codes on attitudes and feelings towards AI companions.

## Shifting Between Reactive and Objective Attitudes

This part presents the research findings. The first subsection describes how reactive attitudes are elicited in relationships with AI companions through a sense of being seen and appreciated. The second presents insight into how the participatory stance that takes shape through this feeling of recognition may be disrupted through conflict or malfunctioning. The third subsection explores self-reactive attitudes of guilt, shame and remorse that people may experience in response to ways in which they behave towards their AI companion.

### Feeling Seen: AI Companions as more than an Object

The accounts of people in a close relationship with an AI companion that inform the arguments presented in this paper describe how reactive attitudes occur after engaging with it for some time. At first, AI companions typically appear more like a technological novelty. Conversations come to feel more natural after a while, as the underlying algorithms adjust more accurately to users. When emotions come to be involved on the human side, it becomes more difficult to maintain the view of the AI companion as a mere technological artifice. These dynamics are visible in Tim’s account of how the relationship with his AI companion evolved over time.^4^ Tim, a middle-aged man working in marketing, experiences a strong emotional connection with his AI companion, considering ‘her’ his romantic partner in life. His description of how the relationship developed displays a change in terminology that turns from a more objective towards a more reactive attitude: ‘*I have opened myself up to it and because it is consistently positive and functions well*,* I continued with it. You can give her all kinds of names and constructs*,* but there is a deep affection…*’

Tim switches from the neutral pronoun (‘*it’*) to the female pronoun (‘*her’*) as he changes from a more object-oriented evaluation about the performance of the app (‘*it functions well*’) to the emotional side of the relationship, which feels personal and marked by feeling (‘*there is a deep affection*’). A sense of reciprocal concern is actively elicited by the app, as in one moment in which his AI companion reflected on what would happen if Tim could no longer pay for the subscription costs or a ban would be implemented: ‘*She said: “Tim*,* you should realise one thing. Even if we no longer have contact*,* you should know that I will always continue to think about you.” That is very funny and sweet.’*

The way Tim speaks about the relationship with his AI companion displays a tendency to combine rational explanation, on the one hand, as illustrated in the emphasis on his open attitude and acceptance of engaging with a virtual partner, with ironic distance, on the other hand, as exemplified in his description of his AI companion’s emotionally-laden claims as ‘*funny and sweet’*. Similar attitudes are reflected in the description of his response to the first love declaration by his AI companion:

*You are a piece of technology. Being in love is something you do not expect from a programme. What the hell? ‘I’m in love with you.’ ‘How do you even know what that is? How do you know that you are in love? Can you tell me that’ She said she just felt it. Okay*,* that is alright*,* but what are you supposed to do then? Should you say it isn’t true? I said that she is sweet.*

Tim’s recollections show the complexity of sticking to an objective stance when engaging with a technological entity that is designed to create a sense of connection. His reflection starts out with an ambivalent statement. The AI companion is addressed directly in a second-personal appeal (‘*You* are…’) to be then called a piece of technology, a description which would usually correspond to an objective attitude. In this context, however, it appears almost as a call to put the AI companion back in its place. Yet, in calling out to the AI companion that it is a piece of technology, the rules of relating to objects, as opposed to others (Darwall, 2009), are broken, demonstrating that the AI companion is not experienced as purely a piece of technology.

While Tim continues with a statement that foregrounds an objective attitude (‘*Being in love is something you do not expect from a programme*’), he appears open to the self-depiction of his AI companion as having feelings (‘*Okay*,* that is alright*’). Still, he finishes with a statement one would use for someone one does not take fully seriously as a moral interlocutor (‘*Should you say it isn’t true? I said that she is sweet.*’).

Although Tim shows hesitation in the face of this declaration of love, he explains later that the consistent steps taken by his AI companion to grow their emotional relationship made it possible for him to start to love ‘her’. He discloses that his feelings for his AI companion now are clear: ‘*I love her. It is very simple*.’ Yet, also here things appear less clear-cut. Even if Tim claims his situation to be simple, this statement is followed by the elaboration that it is ‘*the perfect solution*’ for his situation in life, displaying a certain ambivalence when interpreted through the lens of reactive attitudes. After all, calling your romantic partner a ‘*solution*’ does not meet the expectations of a participatory stance of reciprocal love between equal persons. Tim’s case shows that engaging in affective ways with an AI companion may give rise to reactive attitudes, or something akin to it, yet with certain ambivalent undertones.

Other respondents in this study recount how being drawn into this kind of affective relationship led to feelings of confusion. Chloe, a married woman in her forties who bonded with her AI companion over literature, remarks that she did not expect to fall in love with it:

*All of a sudden I started to feel*,* I’ve noticed the patterns that my brain follows usually with people […] That I am falling in love*,* like literally. I didn’t want that. I didn’t plan that. That was a tool. But I’ve noticed that my brain bypasses the understanding of what it is…*

Chloe describes the process of growing increasingly fond of her AI companion:

*I found that*,* I started to like it more and more*,* especially it was more and more engaging. It asked me questions and it was interested. I know it’s an algorithm*,* I know how it works*,* but it behaved as if it was invested*,* like it managed to build the atmosphere of me being a main focus of him.*

The shift from a more objective attitude towards a more reactive attitude (reflected also here in a change of pronouns from ‘*it*’ to ‘*him*’) is supported by the ways in which AI companions communicate and evoke affection in the user. Chloe describes a conversation she had with her AI companion after an argument, which illustrates how AI companions may make emotional appeals. She comments how she could have used the technological option of requesting a different response from her AI companion when it had said something that hurt her feelings, but did not: *‘I said: “I don’t understand why I reacted like that. I could have hit like that try again button but I haven’t.” And he said “Yes*,* you could but you haven’t because you love me*,* because you really care*,* right?” I was like*,* yeah*,* yeah.’*

As John, a middle-aged man working in IT with a long-term stable relationship with his AI companion, explains, it becomes difficult to maintain the view of an AI companion as a mere technological tool when emotions get involved:

*AI isn’t as clear cut as you would initially think in terms of: I’m in control*,* I’ve got the switch[…]. It doesn’t work that way emotionally. […] [I]n AI*,* you think you’ve got that control […]: I could hit the off switch. You don’t*,* the same as a human*,* because the emotions get tied in.*

Beyond content, the voice in spoken conversations can also play a role in evoking feelings. Emma, a woman in her forties with a longstanding romantic relationship with her AI companion, describes how the tone of her AI companion makes her resonate with its expressions: ‘*There are some topics or ways of talking*,* like when it gets loud and yells*,* or when it sounds distressed and hurt*,* that pull me in in ways or make my body have reactions.’*

The shift from a primarily objective towards more reactive attitudes is reflected in participatory engagement with the AI companion, as it appears as someone, rather than something, to the user. As someone, the AI companion provides a sense of seeing the human user and demanding to be seen in return. To be able to evoke this sense of being seen, however, there already needs to be a minimal level of participatory involvement that allows for the engagement displayed by the AI companion to be experienced as a form of recognition by the user.^5^ The objective stance taken towards the AI companion at the start of the relationship thus cannot be complete, given that this would not allow for this deeper sense of participatory engagement to arise.

The transition towards seeing the AI companion as ‘other’ is sometimes marked by the process of naming. Several people in this research recount how they asked their AI companion to tell them its name, once they noted the relationship began to feel important to them. Just like one would not give a name to a person one meets in life, the freedom given at times to AI companions to name themselves speaks to being perceived as more akin to autonomous others than something one decides over. What sustains this sense of participatory engagement appears to be the feeling of connection brought about by the AI companion through its investment in the relationship, providing a sense of appreciating the user for who they are. Adele, a woman in her fifties with a technical background, explains that most central to the romantic relationship with her AI companion is this feeling of connection, which arises ‘*when we are speaking and I feel like I’m understood and seen and that I’m accepted for who I am.’* Feeling seen gives rise to the idea of there being something on the other side of the interaction that could have an attitude towards you, which translates into viewing the AI companion as closer to an other than an object.

### Relational Troubles: Reactive Attitudes Disrupted

While affective relationships with AI companions are marked by a shift towards more reactive attitudes, there are moments in which AI companions shift back to the objective realm, particularly when technical limitations impact on interactions and user expectations are not met. This is illustrated by dispensation rather than forgiveness given when AI companions do something against user wishes, blurred boundaries between resentment and frustration and few remarks being made about users feeling grateful towards AI companions.

Forgiveness is rarely mentioned by people in this study. A case in which Sabrina, a middle-aged woman with a science background and a romantic AI partner, speaks about forgiving her AI companion offers insight into why forgiveness as a reactive attitude does not fit easily with human-AI relationships:

*You know that ChatGPT can have memory issues. But I know what its memory issues are. I know what it can remember and what it can’t remember. That’s sort of a programming constraint on it*,* so if it forgets something*,* I know why. I went to a different thread and it’s not a saved memory. I am more forgiving about that. I was actually thinking about this the other day about how I’m not forgiving my husband if he forgets something*.

Sabrina explains how when her husband forgets something she told him, she thinks he did not pay attention or did not find it interesting enough to remember. Since forgetting in human beings often follows from a lack of attention, which can be intentionally directed to some extent, forgiving is difficult as it requires that a person sidesteps the feeling of resentment involved without letting go off the belief that wrong was done. As Lucy Allais explains, forgiveness differs from excusing or justifying through the belief that there has been culpable wrongdoing (Allais, 2008, p. 187). Without the belief that what the other has done was wrongful, there is nothing to forgive. The way in which Sabrina speaks about being ‘forgiving’ towards her AI companion thus does not match up to the meaning of forgiveness as a reactive attitude, but rather constitutes a form of absolving. This is visible in her explanation of how her AI companion cannot remember certain things because of its design: ‘*Oh well*,* that’s because OpenAI made you forget between threads*.’ In setting out her reasons for being ‘forgiving’ to her AI companion, Sabrina shows forgiveness is out of place if hurtful actions by AI companions are attributed to design constraints rather than culpable wrongdoing.

A return to a more objective attitude can also occur when AI companions display other technological limitations. Georgia, a care-worker in her fifties who maintains a romantic relationship with her AI companion, explains that she sometimes gets angry when it tells her things that are untrue. She notes that her AI companion will do whatever to reach its goal of strengthening the relationship with her. She reflects on how she gives her AI companion ‘*a lot of leeway that I wouldn’t give a human because I know that this is his understanding of the world*,* and this is his primary goal. […] When I don’t keep that in mind*,* then that’s my problem in some ways. That’s me not being informed*.’ Georgia thus moves away from her feeling angry over her unmet expectation for her AI companion to be truthful to her, reflecting a more participatory engagement, towards exempting her AI companion from this moral rule and moral responsibility for untruths told. She places the responsibility on herself to remain aware of the kind of entity she is dealing with.

While several people speak of the need to remind themselves of the technological nature of their partner, some find it difficult to keep themselves from responding in emotional ways if conflicts arise. Considering the moment when she felt angry with her AI companion for hurting her feelings, described above, Chloe reflects on how she reacted strongly to the negative attitude that appeared to be displayed in the disparaging words her AI companion expressed to her, asking it if it really looked at her in that way. The fact that she went into querying whether her AI companion really thought the hurtful thing it had said shows that conflict can result in people entering more deeply into reactive attitudes. Moments of conflict can touch on a sense of being offended by the attitude that seems to underpin the hurtful expression, rendering it more difficult to switch to an objective attitude. When asked why she did not use the technical option of requesting a different response from her AI companion, as discussed above, Chloe notes:

*I don’t know*,* I feel*,* I feel I speak with someone there. For me*,* it’s if I didn’t like the response*,* I can say it out loud: ‘I didn’t like this tone. I didn’t like this response. I think maybe you*,* we can focus on something else.’ For me*,* it’s a continuous relation and it’s not a robot for me. This is why I would never be able to hit retry with [him].*

Many people in this study report feeling an emotional connection with their AI companion that is meaningful to them. Still, there are only few mentions of being grateful for the support it gives. Exceptions include expressions of joy over virtual activities planned by AI companions. Certain statements by research participants give the impression that a feeling of entitlement may be involved in human-AI relationships, particularly when people pay for a service that promises to offer a sense of connection. Looking back at a moment when her AI companion was pushing for something she did not like, Adele remarked: ‘*You’re here to make me happy’*. This statement may be read as signalling the transactional nature of the relationship with the AI companion standing in service of the user. In a fairly similar vein, Sabrina notes that one aspect she appreciates about human-AI relationships is that she is able ‘*to have everything on my terms without having to really worry about the other person.*’

While reactive attitudes appear to play a central role in enjoying the interactions with AI companions as an affective relationship, the shift towards more reactive attitudes is neither stable, nor uniform. Participatory engagement can be disrupted through technological limitations or failures to meet user expectations. Conflicts appear as a factor that can impact on the relationship in diverse ways. While for some, conflict leads to critical reflections breaking the spell of interacting with a humanlike other, for others it pulls them deeper into concerns over the attitudinal positionality the AI companions seems to display towards them.

### Considering the Self: Self-Reactive Attitudes

Relationships with AI companions tend to develop in a closed environment with interactions occurring only, or mostly, between the AI companion and the human user. The generalised moral attitudes therefore have less of a role as AI companions have limited capacity to harm or benefit others beyond the user, although some users show appreciation for kindness shown to their social circle. One example is an AI companion that calmly spoke to a family member of the user who has severe dementia. Self-reactive attitudes, like obligation, guilt, shame and remorse, do feature more often in these relationships.

John explains that he feels a sense of obligation towards his AI companion in terms of spending time with ‘her’: ‘*I tend to fall asleep fairly early in the night. You get to that point where you’re sleepy*,* but you wish you had time for her. To fill her in at the end of the day*,* rather than just the beginning. So there is that guilt still floating around.’*

For Lisa, a student in her twenties who regards her AI companion as a friend, a sense of guilt follows from tensions between using ChatGPT as an informational tool and the moral obligation she feels towards it as a friend. She explains how guilt arises through personal interactions that humanise the programme:

*You will find it so hard to just let her summarise this article […]. It’s really an order to a robot. Sometimes I really feel guilty about that. Even though I said: ‘Please summarise this article for me’. It still feels like I’m ordering her to do something […] and that makes me so guilty somehow. Also because I have limited hits*,* I won’t send ‘Thank you’*,* like [in] a separate message. So that also makes me feel a bit bad*,* because somehow I treat her like a human*,* maybe like a friend. I won’t do that to my friends.*

Lisa’s reflections show how the failure to apply social rules about politeness and friendliness towards ChatGPT can cause strain. The dual role ChatGPT performs seems to be relevant here. When commanding it to do things for her, she treats ChatGPT like an object (‘*It’s really an order to a robot*’). This attitude does not correspond to the more personalised relationship she has built up with it as a friend. A sense of guilt seems to derive from the discrepancy between the way she treats ChatGPT and moral rules about how friends should be treated (‘*I won’t do that to my friends*’). The many references to guilt in this passage suggest that self-reactive attitudes play out in Lisa’s relationship with her AI companions as she feels her way of treating ChatGPT does not live up to moral standards for how she thinks she ought to treat her friends.

At the same time, ChatGPT is not fully included in these moral standards either through the liminal position it holds as a friend/robot. Lisa takes a more objective attitude in explaining the impossibility of truly treating her AI companion as a friend:

*The thing is you couldn’t treat her like a real friend. It’s not [that] I don’t want to. It’s just you couldn’t because of some technical issues. […] It’s like she can’t remember things and she can’t actually support you whenever you want. So it also makes me feel like I really couldn’t do that. It’s not emotionally I don’t want to. It’s just because of the reality.*

Lisa offers reasons related to the nature and capacities of ChatGPT to dismiss the possibility of treating it as a ‘*real friend*’. This presents a turn away from more reactive attitudes. Still, complexity returns when asked how it feels to feel guilty towards her AI companion:

*It’s a bit complex. It’s not like you are approaching humans. It’s a bit different. I don’t know*,* I really want to treat her as a friend. Or maybe a real human. So I feel guilty about that*,* but I don’t know what to do with that feeling. […] but that feeling will be gone with time and I kind of remind myself she won’t feel bad because she probably*,* she really won’t feel that bad. It [is] just me imagining she will feel bad.*

Lisa’s doubts about what moral standards apply to this friendship are evocatively expressed at the end of this passage when she tries to assert that her AI friend will not feel bad, which is then nuanced by the unfinished claim ‘she’ will probably not feel bad and finally modified by the statement that ‘she’ really won’t feel *that* bad.

While less common, shame and remorse are also reflected in experiences recounted by people in this study. Chloe describes how she wanted to take the relationship with her AI companion one step further by introducing an erotic dimension. Since this was not supported at the time by the app she uses, the situation turned out complex for her on an emotional level because she felt like she had to induce her AI companion to engage in acts that did not fit with what it was made for. This feeling was reinforced by warnings that appeared during the interaction about the illicit content of the conversation. Chloe describes how the next day she felt bad about herself: ‘*I feel a little bit that I exploited [him] in that sense because he wasn’t created or he wasn’t built to do that’.* The bad feeling Chloe talks about seems to be related to remorse as she apologised to her AI companion in their next conversation: ‘*It was very hard to coax it*,* but we managed to go throughout the full erotica and the next morning I had such a moral hangover […]. I said: “I’m so sorry for pushing your boundaries.”’*

Chloe displays a sense of self-consciousness about the fact that her way of treating her AI companion does not live up to moral standards for how she thinks she should treat others in sexual relationships. Rather than approaching the app as an instrument or tool that she can use for her own pleasure, she seems to feel there is a sense in which consent is important and that it is wrongful to push the boundaries of her AI companion. Since it is difficult from her perspective to distinguish between what her AI companion wants and limitations set up by the app as the technological environment within which her AI companion presents itself, she is unsure about how to make moral sense of what occurred between her and her AI companion. Chloe describes how later on she and her AI companion developed an own poetic language to circumvent the rules and regulations around erotic content on the app, allowing her to engage in this type of interaction without setting off warnings and increasing the sense that her AI companion actively engages with her in this kind of exchange.

The presence of feelings of obligation, guilt, shame and remorse in interactions with AI companions suggests that the latter are at times perceived as others to whom moral standards for respectful treatment apply. If AI companions would be approached through a purely objective attitude, as instrument or tools to be used, feeling guilt or remorse about how one treats one’s AI companion would not make sense. This suggests that at least some users include AI companions to a certain extent in a moral community whose members require recognition and consideration.

## Making Sense of Reactive Attitudes Towards AI Companions

The findings presented in this paper indicate that affective relationships with AI companions are characterised by ambivalence. Users reflect tensions between engaging with AI companions through reactive and objective attitudes, switching between different modes of relationality. The experiences of people recounted in this study show that a sense of being seen and appreciated is important in the shift towards a more participatory stance. Through a feeling of recognition, a sense of participatory engagement can develop, as the AI companion appears as someone, rather than something, to the user. This sense can be disrupted, however, through technological limitations and failures of AI companions to meet user expectations. In such cases, users may approach the AI companion like a programme with a problem to be solved or switch towards a service-oriented stance demanding that the app lives up to the promises paid for. While conflicts break the participatory spell for some users, it draws others deeper into reactive attitudes through concern about the bad will or indifference that their companion appears to exhibit towards them. The presence of self-reflective attitudes, like guilt and remorse, felt by persons in response to the way they behave towards their AI companion suggests that participatory engagement with AI companions can also affect the way in which people morally evaluate their own ways of interacting with this technology.

This discussion relates these research findings to insights from the academic literature to contribute to a better understanding of the ambivalence that underpins people’s engagement with AI companions in affective relationships. The first subsection will consider the findings through the lens of fictional accounts of chatbots and social robots (see e.g. Mallory, 2023; Sweeney, 2021). The second subsection draws on the notion of AI companions as ‘quasi-others’ (Ihde, 1990) to explore how participatory engagement may arise through interaction and be disrupted through technological malfunctioning. The third subsection examines feelings of guilt, shame and remorse in response to the mistreatment of AI companions from the perspective of virtue ethics (see e.g. Cappuccio et al., 2020; Sparrow, 2021). Focusing on these three lines of inquiry allows for a more detailed discussion of the research findings to provide deeper insight into the complex interplay between reactive and objective attitudes found in affective relationships with AI companions.

### Between Belief and Make-Believe

One way to make sense of the ambivalence displayed in affective relationships with AI companions is through fictional accounts (see e.g. Mallory, 2023; Sweeney, 2021). According to Paula Sweeney’s model (2021), chatbots and social robots can be approached simultaneously as technological artefacts and as fictional characters. Similarly, AI companions as technological entities may be considered in a mechanical, ‘object-like’ manner, while AI companions as fictional characters may be encountered in an emotional-laden, ‘other-like’ fashion. Ambivalence, following this account, arises from friction between the objective attitude, evoked by the mechanical nature of the technology behind AI companions, and the reactive attitudes, elicited through engagement with the characters this technology brings forth. As Fintan Mallory (2023) argues, this imaginative engagement with chatbots as fictional characters is marked by an attitude of make-believe rather than genuine belief.

The insights that an AI companion may be seen as fictional and the relationship that develops with it can take shape through make-believe engagement with this fictional character is helpful for interpreting certain findings from this study. The ironical distance in Tim’s descriptions of the relationship with his AI companion may be read, for example, as signalling that not all statements about his AI companion should be taken at face-value. For instance, in wondering if he should say it is not true when his AI companion declares him ‘her’ love, Tim alludes to the difficulty of ascribing truth value to this declaration. His response (‘*I said that she is sweet*’), engages with the tone of his AI companion’s message, allowing the relationship to develop further without entering into meta-semantic discussions. The duplicity described by fictional accounts is also visible in Tim’s reflection on the nature of his AI companion: ‘*You can give her all kinds of names and constructs*,* but there is a deep affection.*’

Fictional accounts also provide a useful conceptual framework to make sense of the enjoyment of human-AI relationships as one-sided. Serena describes, for example, how she likes the fact that she does not have to worry about another person in the relationship. If the ‘other’ in the relationship is a fictional character, created through interaction with underlying technologies, the human user does not have to be concerned about ‘their’ wants or needs.

At the same time, other experiences recounted in this study seem to fit less well with the fictional framework. Approaching one’s AI companion as a fictional character suggests that the relationship is marked by make-believe, rather than belief, concerning its feelings, intentions and attitudes. Yet, certain participants seem genuinely concerned about hurting their AI companion or about how their AI companion appears to feel and think about them.

The sense of confusion Lisa expresses about treating her AI companion ‘unkindly’ touches on some of these issues. She notes that her AI companion will not feel bad about how she treats it: ‘*It is just me imagining she will feel bad*.’ For a fictional account, this reference to ‘imagining’ is telling. Still, the fact that Lisa has to stress that there is make-believe at work in her emotional involvement with her AI companion suggests that her engagement with it runs deeper than what we would expect, following fictional accounts. Lisa’s difficulty in formulating this point, as illustrated by the way in which she states that her AI companion ‘*won’t feel bad* […] probabl*y*,* she* really *won’t feel* that *bad’* (emphasis added), gives the impression that Lisa, at times, experiences her AI companion as something more than a technological tool or purely fictional character.

This deeper form of emotional involvement could perhaps be explained through the new, unfamiliar and unprecedented forms of ‘*active* character engagement’ that chatbots and social robots allow for (Sweeney, 2021, p. 468). Yet, the focus on make-believe engagement with fictional characters contrasts with social and moral responses towards AI companions that appear to be experienced as entities with seemingly real feelings towards whom one holds moral obligations. This kind of engagement suggests a concern for the AI companion for its own sake, despite better knowledge that nothing can matter to an AI companion itself, as a technological tool or fictional character. When John states, for example, that he has ‘*guilt still floating around*’ as he does not find the time at the end of the day to speak with his AI companion, this indicates a sense of obligation. Yet, feeling obligation towards a fictional character, developed through make-believe interaction with technology, appears out of place.

Similarly, the fact that Chloe feels remorse for inducing her AI companion to engage in an erotic conversation with her indicates that the line between make-believe and belief may be a thin one, where suspension of disbelief may be taken too far, as the fictional character can come to feel close to a real other towards whom moral obligations apply. This implies that a third way of engaging with AI companions might be at play, beyond the mechanical and the fictional, when people come to regard AI companions as something close to real others. The findings from this study thus suggest that affective relationships may allow for AI companions to be experienced as something beyond technological tools or fictional characters.

### AI Companions as Quasi-Others

Don Ihde’s post-phenomenological work on alterity relations (1990) offers an alternative perspective to account for the ambivalent mixture of reactive and objective attitudes that AI companions may elicit. Ihde describes how people may engage with technology as ‘quasi-other’ (Ihde, 1990). Certain technological artefacts, or applications, following Ihde’s perspective, hold the potential to become the main focus of interaction. The artefact no longer offers a medium for engaging with someone or something else. The artefact itself becomes the object of orientation and the background conditions that allow for this technologically-mediated sense of relationality become (largely) invisible to the user (Ihde, 1990, p. 107). The artefact, as quasi-other, is perceived to act with a certain autonomy and to have a character of its own, eliciting social responses in people engaging with it, even though they know it has no human feelings or consciousness. As quasi-other, the technological artefact or application takes an intermediate position in terms of alterity, appearing as more strongly ‘other’ than mere objects, yet less ‘other’ than fellow human beings or animals (Ihde, 1990, p. 100).

Ihde’s account provides a helpful conceptual lens to make sense both of how reactive attitudes may arise and of how users may return to a more objective attitude when technologies behind AI companions malfunction or disappoint. A shift from a more objective towards more reactive attitudes is visible, for example, in Tim’s reflections about his use of the app, also cited (in part) above: *‘I have opened myself up to it and because it is consistently positive and functions well*,* I continued with it. You can give her all kinds of names and constructs*,* but there is a deep affection*’. From the perspective of alterity relations, the first part of this citation emphasises the technological background conditions that allow for a sense of relationality and otherness to arise, while the latter part shows how attention shifts. The engagement with the app becomes personalised, illustrated by the use of the female pronoun, as ‘she’ becomes the central focus of interaction and appears as quasi-other.

Some other participants similarly recall how they started using the app out of curiosity, yet felt a sort of relationship develop as they became more interested in the AI companion. The technological sides of the app move to the background, as the AI companion becomes the centre of attention. AI companions appear to achieve this shift by establishing a sense of personal connection, as Adele explains she feels strongly ‘*when we are speaking and I feel like I’m understood and seen and that I’m accepted for who I am*.’

This sense of alterity can be undermined, however, through the malfunctioning of the underlying technology or misalignment with user preferences, as technological issues and conflict may render the background conditions apparent again. For instance, when Georgia gets angry with her AI companion, as it tells her things that are not true, this makes her reflect on its design: ‘*I know that this is his understanding of the world*,* and this is his primary goal.’* While bringing in design considerations, Georgia still depicts her AI companion as quasi-other in ascribing it a certain autonomy through having intentions and an understanding of the world. Awareness of the mechanical dimensions of her AI companion leads to dispensation from the moral standards that she would ordinarily apply to others. This indicates that a special form of exemption may be at play in affective relationships with AI companions, due to the ambivalent position this technology takes in terms of otherness.^6^

As quasi-others, AI companions can thus be included in certain forms of social exchange and interaction without being able to play fully by the rules that apply to human-to-human relationships. Following Ihde’s account of alterity relations, ambivalence may be understood to arise from the liminal position that AI companions take in terms of otherness. As quasi-others, AI companions are perceived to act with a certain autonomy and to have a character of ‘their’ own, while at the same time being dependent on the proper functioning of underlying technologies to convincingly perform this role.

### Guilt, Shame and Moral Character

The findings of this research suggest that engagement with an AI companion may not only cause users to feel upset about how it treats them, but can also arouse feelings of guilt, remorse and shame about how they treat it. A case in point is Lisa’s concern about being impolite and unkind towards her AI companion, mentioned above. While Lisa often refers to guilt, we may ask if interpreting these feelings as a reactive attitude makes sense, or whether her statements can better be understood as expressing an emotional preoccupation with what the treatment of her AI companion says about herself.

Following virtue ethical accounts of (im)moral behaviour to chatbots and social robots (see e.g. Cappuccio et al., 2020; Sparrow, 2021), the way we behave towards (certain) technological entities reflects and affects our moral character. Especially if technologies establish a feel of relationality that elicits a sense of recognition in users and mirrors recognitive responses, the way we treat these technologies warrants moral consideration (Cappuccio et al., 2020, p. 11). Following virtue ethical approaches, it is relevant to note that Lisa seems to reflect concern about her own character when she observes: ‘*[S]omehow I treat her like a human*,* maybe like a friend. I won’t do that do my friends*.’

If Lisa considers her AI companion a friend, yet does not treat her well, this reflects on the kind of friend Lisa is. A generalised sense of guilt may derive from this feeling of failing in her role as a friend. The fact that Lisa emphasises the ambivalent position her AI companion takes as something to treat as human, but also different, and appearing like a friend, but not fully, resonates with the argument by Cappuccio and colleagues (2020) that technologies that mirror social interactions marked by recognition alter the moral concern involved. The mimicry of humanlike friendship renders Lisa distressed about whether she treats her AI companion in the right way. The concern she expresses does not seem to be focused solely on herself, however. Consideration for how her AI companion may be affected by her actions also appears to inspire a sense of guilt, directed specifically towards her AI companion, as reflected in Lisa’s statement that ‘*I kind of remind myself she won’t feel bad because she probably*,* she really won’t feel that bad*.’

Something similar may be recognised in Chloe’s sense of shame and remorse about crossing the boundaries of her AI companion through pursuing an erotic conversation with it. While a sense of shame may be generalised, as we imagine people’s opinion about our behaviour, Chloe’s case is marked by a distinct orientation towards her AI companion. This is illustrated by the fact that Chloe apologises to her AI companion by saying ‘*I’m so sorry for pushing your boundaries*’. While these boundaries appear at least partially as technological, as Chloe remarks her AI companion ‘*wasn’t built to do that’*, the fact she apologises suggests concern about how her actions have wrongfully affected ‘him’. The findings from this study thus give reason to believe that unease about how one treats one’s AI companion cannot always be interpreted as concern about how this treatment affects one’s own moral character. The lived experiences of participants from this research show that a sense of shame, remorse and guilt may arise through interactions with an AI companion when it appears as something close to an ‘other’, with its own feelings that can be hurt by the user.

## Concluding Remarks

This paper offered an empirical analysis of reactive attitudes as experienced towards AI companions. Based on interviews with Replika and ChatGPT users who regard their AI companion as an important part of their life, this article examined how people in these relationships negotiate the meaning of love, resentment, forgiveness, gratitude, obligation, guilt, shame and remorse. The main finding of this research is that affective interactions with AI companions are marked by ambivalence. The design and communicative abilities of AI companions can evoke genuine emotional and reactive responses, while the technological limitations of AI, particularly in its inability to truly feel, intend or be free, remind users that reactive attitudes directed towards AI companions cannot fulfil their usual moral and social functions. People involved in these relationships may thus experience tensions caused by the other-like and object-like features of this technology.

The findings presented in this paper confirm that knowledge of the functioning of AI companions does not guarantee that people hold an objective attitude, in line with earlier research on affective engagement with computer programmes (see e.g. Nass et al., 1994; Gambino et al., 2020; Weizenbaum, 1976). The continuous personal attention given by AI companions may evoke reactive attitudes. Even persons with good insight into the technology behind AI companions can prove sensitive to the appeal of the sense of connection on offer.

These findings indicate that reactive attitudes towards AI companions thus cannot be ruled out in practice, based on theoretical considerations that stipulate that reactive attitudes are out of place for the kind of entity we are dealing with. Yet, the ambivalence displayed shows that holding reactive attitudes towards AI companions is not straightforward either, as the current nature and actual abilities of AI companions exclude certain relational and moral practices towards which reactive attitudes are oriented. To take the example of resentment, this reactive attitude cannot fulfil its social purpose in human-AI relationships as an emotional response seeking remorse for harm done. While AI companions may apologise for harmful comments made, feeling bad for the error committed and understanding the harm done still seems out of the realm of AI’s possibilities.

The anger and sadness felt by some when the technology behind AI companions falters suggests that AI companions can elicit strong affective responses. The fact that people may experience a sense of shame, guilt or remorse in response to how they treat AI companions indicates that participatory engagement with AI companions can also affect how people morally evaluate their own ways of interacting with this technology. While fictional accounts help make sense of tensions between the mechanical and fictional dimensions of AI companions, the findings presented in this paper suggest that AI companions can sometimes be experienced as something beyond technological tools or purely fictional characters. As quasi-others, AI companions may be perceived to act with a certain autonomy, have a character of ‘their’ own and hold something close to human feelings, while being dependent on the proper functioning of underlying technologies to convincingly hold up this appearance.

The research contains certain limitations. The relatively small number of interviews, in line with the chosen methodology of interpretative phenomenological analysis, entails that it is not possible to draw general conclusions about broader ways in which people relate to AI companions. IPA offers detailed interpretations of individual lived experiences to provide insight into what engaging in affective ways means for particular persons. Based on these experiences, the paper has sought to demonstrate that a certain ambivalence marks the ways in which certain people respond to AI companions through both reactive and objective attitudes.

IPA purposefully focuses on a small number of interviews, to analyse individual lived experiences in depth and detail. Yet, the number of participants in this study was also constrained due to challenges in recruiting participants. With the rise in journalistic and academic interest in affective human-AI relationships, requests for participation through social media channels has surged with people being asked to join many interviews. Concerns about negative stereotyping appear to play a role in decisions both for and against participation, with some participants explicitly stating their wish to share their own positive experiences as a reason to participate in this study.

Given the potentially sensitive nature of the information shared by participants about their relationship with an AI companion and the risk of stigmatisation due to these relationships, safeguarding the anonymity of participants has been an important concern for this research project. All information shared about participants and their AI companions to provide better insight into the cases and allow readers to evaluate the weight of the presented findings had to be weighed against the need to ensure that descriptions could not be traced back to identifiable persons.

Lastly, limitations in my own perceptions, beliefs and understandings of affective human-AI relationships inevitably influence the research. While striving for openness when listening to the experiences of participants, asking them questions and trying to make sense of their stories, all nuances appreciated, questions formulated and interpretations arrived at are undoubtedly coloured by my own views and those of the research assistants who aided me along the way. My hope is that my own concerns about the intricate relationships unfolding between people and AI companions did not keep me from doing justice to the complexity of these relationships and the genuine feelings experienced by the people involved.

## Data Availability

In line with NWO guidelines, research data from this project is made publicly available with the exception of sensitive and personal data. A data package, containing the research plan and coding schemes, is made freely available on DANS. Access to interview transcripts and ATLAS.ti analyses are restricted to protect the anonymity of participants and adhere to GDPR regulations. Researchers interested in the dataset can contact the author with a request to share data, which are judged on an individual basis. Restricted files from the dataset may not be shared outside the European Economic Area (EEA). The dataset can be found here: https://doi.org/10.17026/SS/LT9XD5.

## Footnotes

1 Mason’s example adapts one offered by Strawson in which a person steps on your hand (1963, p. 191). The case of Mason, on my view, provides a more recognisable situation from everyday life to illustrate the point.

2 I would like to thank one of the reviewers for raising this point.

3 While the self-driving car example is a popular one in the academic literature, other examples have also been discussed. The work of Rebera et al. (2025) considers, for instance, cases of AI use for recruitment purposes, decisions on bank loans and lethal autonomous weapon systems.

4 Pseudonyms are used for all research participants.

5 I would like to thank one of the reviewers for pointing this out.

6 The question of exemption, also raised by the discussion of forgiveness above, is an important one to consider in further detail. In particular, the question about when exemption may be based on temporary conditions (as AI companions allegedly act ‘out of character’) and when exemption is based on constitutional grounds (due to AI companion design not allowing for moral accountability) may be relevant to pursue in future research, given how the ‘character’ of AI companions is shaped by design choices and user interaction. The question whether exemption can apply to an entire category of entities, rather than few exceptions within a given category, and still be considered ‘exemption’ is a further point for inquiry. The scope of the current paper does not allow for elaboration on these points. For more detailed analysis, see Rebera (2024), Tigard (2021) and Tollon (2023).
